# Saracura-Mirá, a Proposed Brazilian Amazonian Adaptogen from *Ampelozizyphus amazonicus*

**DOI:** 10.3390/plants11020191

**Published:** 2022-01-12

**Authors:** Suzana Guimarães Leitão, Gilda Guimarães Leitão, Danilo Ribeiro de Oliveira

**Affiliations:** 1 Faculdade de Farmácia, Universidade Federal do Rio de Janeiro, Av. Carlos Chagas Filho, 373, Bl. A2, sl. 10, Rio de Janeiro 21941-902, Brazil; 2 Instituto de Pesquisas de Produtos Naturais, Universidade Federal do Rio de Janeiro, Centro de Ciências da Saúde, Bloco H, Rio de Janeiro 21941-902, Brazil; ggleitao@ippn.ufrj.br

**Keywords:** amazon adaptogen, traditional medicine, tonic plants, fortifier plants, depurative, saracura-mirá, cervejinha, cerveja de índio, cerveja de preto, Amazonian ginseng

## Abstract

The Amazon Forest is known all over the world for its diversity and exuberance, and for sheltering several indigenous groups and other traditional communities. There, as well as in several other countries, in traditional medical systems, weakness, fatigue and debility are seen as limiting health conditions where medicinal plants are often used in a non-specific way to improve body functions. This review brings together literature data on *Ampelozizyphus amazonicus*, commonly known in Brazil as “saracura-mirá” and/or “cerveja de índio”, as an Amazonian adaptogen, including some contributions from the authors based on their ethnographic and laboratory experiences. Topics such as botany, chemistry, ethnopharmacological and pharmacological aspects that support the adaptogen character of this plant, as well as cultivation, market status and supply chain aspects are discussed, and the gaps to establish “saracura-mirá” as an ingredient for the pharmaceutical purposes identified. The revised data presented good scientific evidence supporting the use of this Amazonian plant as a new adaptogen. Literature data also reveal that a detailed survey on natural populations of this plant is needed, as well as agronomical studies that could furnish *A. amazonicus* bark as a raw material. Another important issue is the lack of developed quality control methods to assure its quality assessment.

## 1. Amazonian Medicinal Plants Used as Fortifiers and Tonics and the Relationship with Adaptogens—The Case of “Saracura-Mirá”

Throughout history, it is possible to see that in traditional medical systems in various countries or regions of the world, signs and symptoms such as weakness, fatigue and debility are seen as limiting factors for health conditions. In the books of the Old Testament Bible, it is possible to see mentions that the frailties emanate from physical and mental illnesses installed in the body, related to the understanding of the disease and suffering of the time [1]. In this context, according to Oliveira and Leitão [2] medicinal plants are often used in a non-specific way to improve body functions. The use indications of these plants, often mentioned in ethnobotanical or ethnomedical research, are diverse [3,4]: aging, Alzheimer’s disease, bad memory, burnout, dementia, despondency, difficulty in reasoning, diseases caused by nervous exhaustion, fatigue, forgetfulness, general debility, infertility, lack of attention, laziness, listlessness, loss of memory, memory weakness, old age, organic weakness, physical and mental exhaustion, physical or intellectual weariness, physical or sexual debility, poor memory, feeling run down, senility, sexual impotence, sexual weakness, stress, tiredness, to improve health, weak head, weakness of nerves, unwillingness, etc.

In such cases, people often turn to tonic, rejuvenating and restorative plants, especially when a reduction in muscle function, cognition, sexual performance or resistance to infections is noticed. These adversities are more pronounced during aging because this process leads to a progressive loss of capacity [5]. However, they can happen, regardless of age, in cases of weakness that are caused by infectious diseases or by stress or psychosomatic problems.

When we seek to transpose these traditional uses to evidence-based medicine, adaptogens stand out. The definition of the term has been evolving since it was coined by Nikolai Lazarev, in 1958, in the former Soviet Union, to classify a group of substances that can improve the body’s non-specific resistance after being exposed to various stressful factors, promoting a state of adaptation to the exceptional situation [6,7]. The term adaptogen is currently widely used in integrative and complementary medicine, and a recent review on the updates of the adaptogen definition has been released, incorporating the increasing body of scientific evidence related to understanding their pharmacological and molecular mechanisms of action [5]. In this context, some plants already consecrated today as adaptogens can be highlighted such as Korean ginseng (*Panax ginseng*), Siberian ginseng (*Eleutherococcus senticosus*), ashwaganda (*Withania somnifera*), gomishi (*Schisandra chinensis*) and artic root (*Rhodiola rosea*) [5,8].

The Amazon Forest is known all over the world for its diversity and exuberance, and for sheltering several indigenous groups and other traditional communities such as *seringueiros* (rubber tappers), *ribeirinhos* (riverside fishermen) and *quilombolas* (maroons), who have learned about and with the forest through centuries (or even millennia) of observation and experimentation. The term *quilombola* (*quilombolas*, in the plural) was coined to designate the fugitive black slaves of the West Indies and Guiana in the 17th and 18th centuries and also a descendant of such a slave. In Brazil, by definition, *quilombolas* are the remnants of the quilombos, or descendants of the black slaves brought from Africa to Brazil during the sixteenth to eighteenth centuries to work on the farms. More recently, the term has gained a broader meaning, and it refers to: “… groups that have developed resistance practices in maintaining and reproducing their characteristic lifestyles in a particular place” [9]. The term quilombola comes from the Tupi-Guarani *cañybó* which means “the one who escapes a lot” [10]. In these traditional communities, the concepts of disease and infirmity exist in distinct contexts—for example: the diseases of the spirit and the diseases of the body [11], the natural diseases and the unnatural ones [12]. In the case of diseases of the spirit or natural diseases, there is a strong influence of the representations of the natural world and the forces that govern it, representations of the person and, finally, the forms of relationship between the human world, natural world and the supernatural world. Any interpretation of the disease is thus immediately placed in the totality of its socio-cultural frame of reference [13]. Toning and cleansing the body are fundamental strategies to resist (or prevent) and treat disorders of any nature, including those of a physical and spiritual nature, targeting health and well-being, in a holistic context.

Several South American and African plant species have stood out more recently as “new” adaptogens, such as the Peruvian maca (*Lepidium meyenii*) and Yohimbe (*Pausinystalia johimbe*), but it is worth mentioning three emerging Amazon medicinal plants for their adaptogenic potential, namely marapuama (*Ptychopetalum olacoides*), cat’s claw (*Uncaria spp*.) and “saracura-mirá” (*Ampelozizyphus amazonicus*), plants that have historically stood out as tonics, due to numerous specific and unspecific indications for use, and for their reputation far beyond the Amazon territories [2]. The latter, *A. amazonicus*, has attracted our attention for being a fundamental plant used by *quilombola* communities of Oriximiná, in the north of Brazil in the Amazon region, especially used as a tonic and fortifying agent, as well as to treat and prevent malaria [14].

Therefore, ethnopharmacological evidence that supports *A. amazonicus* as an adaptogen is discussed more deeply in this review, including botany, management, conservational and vegetal production aspects, informal trade and ethnobotanical evidence that supports the plant as an adaptogen as well as the pharmacological evidence and chemical composition, encompassing the gaps and future perspectives to transform this medicinal plant into an established herbal medicine.

## 2. Botany, Management, Conservational and Vegetal Production Aspects

**Figure 1:**
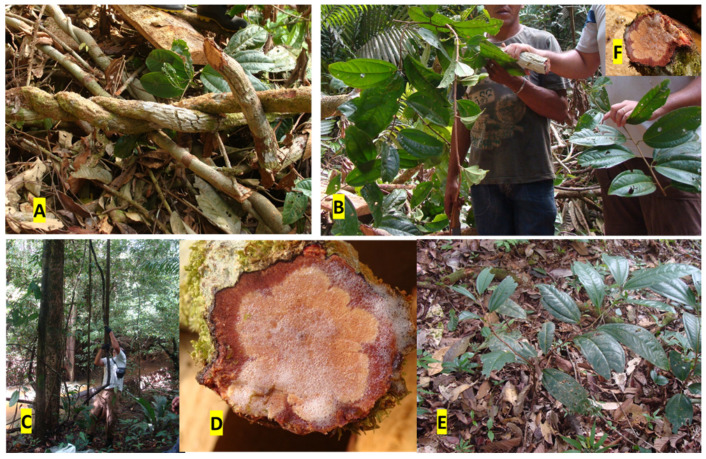
*Ampelozyziphus amazonicus*. (**A**) Characteristic robust woody liana with twisted stem; (**B**) plant collection showing details of the alternate opposite simple leaves on young stems; (**C**) habitat and habit of *A. amazonicus* in the forest; (**D**,**F**) transversal cut of the liana showing the brownish red ritidome with whitish sapwood that produces abundant foaming (saponin rich) upon cutting; (**E**) seedlings of *A. amazonicus* in a terra firme area. Photos by S.G. Leitão, taken at Ilha do Macaco, along the Erepecuru River, Brazil.

*Ampelozizyphus* is one the fourteen genera of the Rhamnaceae family occurring in Brazil as reported in the Brazilian Flora [15]. The genus was described in 1935 by Adolphe Ducke (1876–1959) based on specimens collected in Manaus at the Mindú and the Tarumã river waterfalls in Amazonas State [16]. Etymologically, when *Ampelozizyphus* was first described by Ducke, it was allied with *Ziziphus* Mill. (Rhamnaceae) because of the immature berry-like fruits that were known at the time [17] and it was included in the Zizypheae tribe. More recently, based on molecular phylogeny, the new tribe Ampelozizypheae was created to better accommodate the genus [18]. Nowadays, the genus includes three species but, until 2008, it was considered monotypic, with only a single species—*Ampelozizyphus amazonicus* Ducke (Figure 1)—characterized by a high-climbing lianoid habit, occurring in the *terra firme* lowland forests (altitude range of 50–300 m) of the Brazilian Amazon region, in the states of Amazonas, Pará and Roraima [16], as well as in Peru, Colombia, Venezuela, Guyana, Suriname and French Guiana [17].

In 2008, a second *Ampelozizyphus* species was discovered and described as a small tree (5 to 10 m) during the exploration of the cloud forest flora of the Venezuelan Coastal Cordillera around Cerro La Chapa in Yaracuy State and in the Ávila mountain range in Miranda State, at altitudes of 300–600 m, namely, *A. guaquirensis* Meier & P. E. Berry. It grows on lower mountain slopes and along rivers, flowering in July and August, during the rainy season and mature fruits are produced during the dry season from November to April [17].

More recently, a third species with an arboreal habit (6 to 10 m tall) has been described and named *A. kuripacorum* Aymard & Castro-Lima, occurring in the upper Cuyari River in Guianía Department, Colombia. The new species grows at the margins of flooded forests drained by the black water river Caño Guaviarito, an affluent of the upper Cuyarí River, Colombia, at altitudes of 100–300 m [19]. *A. kuripacorum* is named after the Kuripaco indigenous people, a nation that has lived for centuries in the upper Rio Negro area [19].

Despite the finding of the two arboreal species of *Ampelozizyphus* in the Amazon region of Colombia and Venezuela, currently, only *A. amazonicus* is described in Brazil, with a lianoid habit. The plant is a robust liana (Figure 1A,C), without tendrils, with a cylindric, striated and rusty-colored stem, with brownish lenticels (Figure 1A,B). The stem, when cut, produces foam due to the massive presence of saponins in the plant (Figure 1D). The leaves are large, alternate, petiolate, with ovate to oblong contour, and coriaceous consistency (Figure 1C,E). Its blade (10–22 cm × 6.2–11 cm) presents a round or obtuse base, acuminate apex, entire revolute margin, with prominent veins (three to five) on both surfaces [18,20]. It blooms from October to December and fruits from November to February [15]. Detailed macroscopic and microscopic morphodiagnostic characters have been described [20] for the leaves (not medicinally used) and secondary root, but not for the stem bark, the main plant part used in traditional medicine.

The plant is sold throughout the Amazon region, but is not commercialized in the south, southeast or midwest states of the country. However, it is possible to acquire it through internet sites, but apparently all the material comes from extractivism. Studies concerning the ecology and management of *A. amazonicus* are scarce. Obase [21] surveyed the natural populations of *A. amazonicus* in a rural community of Presidente Figueiredo (Amazonas State) and the factors influencing its density in the forest such as percentage of canopy cover and leaf area index. Results showed that density is negatively influenced by the percentage of canopy cover, which is consistent with lianoid plants that depend on a greater availability of adequate supports to reach the forest canopy. The work of Siqueira [22] evaluated the occurrence of natural populations of the plant along the Solimões and Amazonas Rivers, as well as conservation strategies by in vitro techniques since the plant is commonly used by populations in the collection areas, which are under the anthropic action of deforestation. The use of different carbohydrate sources in the in vitro conservation of *A. amazonicus* influences the height of the aerial part. With increasing concentrations of sucrose there is a tendency to increase in height and the number of buds, but the species shows very slow in vitro growth [22]. The addition of mannitol and sorbitol to the culture medium is unsuitable for in vitro conservation of *A. amazonicus* microcuttings due to low rates of survival and oxidation of the microcuttings.

Viana Junior [23] reported works on sexual and asexual propagation of wild and farmed plants of “saracura-mirá”, with four concentrations of indole butyric acid. *A. amazonicus* can be propagated by seeds, a fact shown by the large number of young individuals around the adult plants, and by cuttings [23]. Young plants can be acclimated for cultivation as long as the requirements regarding soil, temperature, light and humidity are met. Plants of *A. amazonicus* acclimated and transferred to different environments showed from 88% to 100% survival during the research period, indicating that they can be cultivated outside the natural environment. The seeds showed a high germination power, with values above 98% in washed sand substrate. In asexual propagation, the plant was able to reproduce by layering and the method of wire strangulation showed 53% rooting. In the cuttings, the species had a low rooting rate (<12%), requiring further studies with other sources of plant regulators and a longer time for evaluation.

These studies summarize the present status of the knowledge about conservation and cultivation, showing the latter could be an option to overcome a future superexploitation of the natural populations of the plant, something that can gain even greater dimensions due to its growing reputation among the population, which will be highlighted in the ethnobotanical and informal trade data below.

## 3. *Ampelozizyphus amazonicus* Ducke as an Adaptogen: Ethnobotanical and Ethnopharmacological Evidence

*A. amazonicus* is an Amazonian medicinal plant popularly known as “saracura-mirá”, “saracura-corá”, “saracura-muirá”, “saracura”, “curupira-mirá”, “paa-camiuu-ho”, “cerveja-do-mato” (bush beer), “cervejinha” (little beer), “cerveja-de-índio” (indian beer) or “cerveja-de-preto” (quilombola beer), “cerveja da Amazônia” (Amazon beer), “viagra da amazônica” (Amazon viagra), Ijo Sev (Ikólóéhj ethnicity) and xiwiriati (Wai-Wai ethnicity) [14,24,25,26].

Several ethnopharmacological studies conducted in the Amazon region have shown that the plant is used as a stimulant and tonic [24,27], as well as a fortifying agent and aphrodisiac [3,28], in addition to many other indications for the use of the species. An aqueous drink with reported tonic, aphrodisiac and antimalarial properties can be prepared from the bark and roots of the plant [27,28,29].

The medicinal use indications of *A. amazonicus* with direct or indirect relation to adaptogen potential were raised by a literature survey and are summarized in Table 1, based on 33 references where 52 distinct indications of use were identified. The use indications were categorized into the following adaptogen profiles as: immunostimulant agents (promote improved resistance against bacterial and viral infections, and also against chronic inflammation), nootropic agents (improve brain functions such as memory, learning, perceptual ability and reasoning power and concentration), anabolic agents (activate metabolism, increasing the synthesis of nucleic acids, proteins and, therefore, as growth-promoting agents), tonics (improve lack of tone and debility of the body as a whole, or in specific organs, acting to improve physical “performance”) and geriatric agents (assist in the prevention of diseases or symptoms related to age), indicating in parentheses the number of references (primary sources) that have citations for each of the mentioned indications. When analyzing the use indications for *A. amazonicus* according to the supposed adaptogen profiles, the highest number of citations (found in the references) was for immunostimulant agents (49), followed by tonic/geriatric (32), anabolic (3) and nootropic (2) agents, respectively, with some overlapping indications, especially immunostimulant and tonic/geriatric agents. Considering that plant adaptogens should have at least one of the properties highlighted in Table 1, the present literature data survey shows the broad adaptogen profile of *A. amazonicus*, so as to propose it as an “Amazonian ginseng”. In summary, based on ethnopharmacological surveys, the plant can be categorized in all adaptogen profiles.

In an ethnopharmacological study carried out with local specialists from the *quilombola* communities of Oriximiná, located in the Amazonian state of Pará, Brazil, *A. amazonicus* stood out as one of the most important plants among 254, representing a vital element for their survival in the Amazon Rainforest over the centuries [3]. Analyzing the adaptogen concept in the light of the lifestyle of these *quilombola* communities, one can highlight the medicinal uses of *A. amazonicus*. These people live far from the urban centers, deep in the forest, since the beginning of the 19th century, basically living on fishing and hunting, extracting edible fruits and small-scale banana and cassava farming, as well as the extractivism of medicinal and food plants such as the Brazil nut, among others. They are in full contact with nature, in a remote region with high biodiversity, which is why their knowledge of medicinal plants is so rich, coming from the mix of a strong African matrix associated with the knowledge of Portuguese and Amerindian colonizers. As these *quilombola* are “castanheiros”, that is, Brazil nut collectors, they have always been exposed to various tropical diseases and need strength to transport the heavy bags of collected nuts. Therefore, *A. amazonicus* became one of their greatest allies [3,29].

**Figure 2:**
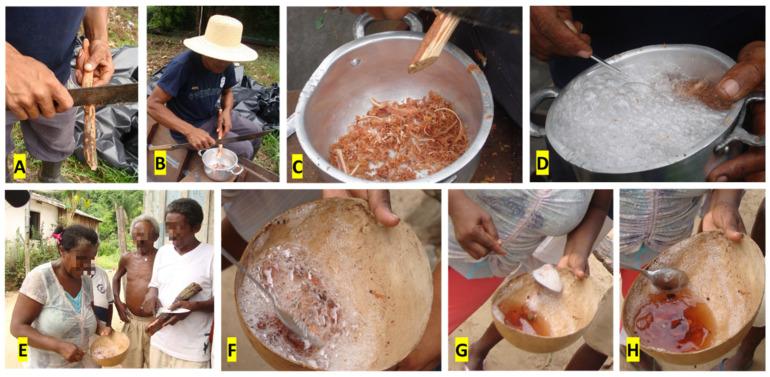
Preparation of the “saracura-mirá” drink. (**A**) Cleaning a piece of vine; (**B**,**C**) the bark is scraped into a bowl; (**D**–**F**) it is vigorously beaten with water, until abundant foam is formed; (**G**) the foam is removed and discarded. This process is repeated seven times; (**H**) the drink is ready after about 10 min of preparation.

Le Cointe [30] possibly brought the first written account of the plant, in which he says that “the Indians use the sap of the root against marsh fever (malaria); has a taste for beer; and a sparkling drink is obtained by tapping the new stems in water”. The traditional *quilombola* method of obtaining the “saracura-mirá” drink consists of a quick and efficient cold extraction (Figure 2), which enables its immediate preparation in the forest, while parts of the stems and/or roots are taken as a powerful and emergency remedy for people dwelling in the community. Possibly, this method could be similar to the one used by the indigenous people from whom the *quilombola* learned to use the plant. Oliveira et al. [14] highlights that, initially, there is a process of weeding/cleaning a piece of vine, and then the bark is scraped into a bowl, in which it is vigorously beaten with water, until abundant foam is formed, which is removed and discarded. This process is repeated seven times, until the drink is ready after about 10 min of preparation. No qualitative differences in the chromatographic profiles were found when comparing the bark decoction or maceration, as well as the drink obtained in the traditional method and the foam which is discarded in the preparation [14]. Furthermore, some *quilombola* also make tea from the wood of the remaining vine (without the bark) and from the leaves and consume them, claiming that these also have the same effects, but weaker than the bark. This report demonstrates a potential for the use of other plant organs, which would contribute to better use and conservation of the species, even if the species were to be cultivated and domesticated.

Considering the immunostimulating/adaptogen profile of “saracura-mirá” among the *quilombola* of Oriximiná, it is worth highlighting its use as a powerful drink in the treatment and prevention of various diseases, being the main and fundamental plant for malaria, without any need to differentiate its use as a prophylactic and/or curative [29]. Several historical data and ethnobotanical/ethnopharmacological studies have corroborated these uses in several places in the Amazon (Table 1). There are also some studies that show “saracura-mirá” as one of the main antimalarial plants [45,46,47], as well as the work of Léda [53] in which there was a high agreement of use categories in the literature about Amazonian medicinal plants for “saracura-mirá” regarding “infectious and parasitic diseases” related to the International Classification of Diseases (ICD-10, https://icd.who.int/browse10/2010/en, accessed on 13 September 2021), similarly to the predominance found here of the plant as an immunostimulant agent in the treatment and prevention of malaria. Recently, the medicinal potential of *A. amazonicus* has gained more strength due to the fact that “saracura-mirá” is being used in the prevention and treatment of COVID-19 by traditional Amazonian communities [49,54].

The second principal adaptogen profile of the plant is related to tonic and geriatric agents of *A. amazonicus*, widely used as a tonic in general, giving energy, revitalizing, rejuvenating and fighting fatigue and indisposition, in addition to acting as a tonic for the organs, including the liver (possibly acting as a cholagogue/choleretic, also considering the bitter characteristic of the plant), the stomach and intestine (for lack of appetite and as a purgative tonifying the digestive system), the kidneys (diuretic) and as a depurative (helping all organs in debugging and cleaning the body). *A. amazonicus* is also used by these traditional groups to harmonize the central nervous system in combating insomnia/sleep disorders and other mental problems especially related to stress, and it is used as aphrodisiac, it being reported that “whoever takes ‘saracura-mirá’ can have many children” [29].

The plant is also used for improving the memory, meaning that it might act as a nootropic agent. A more appropriate *quilombola* term to describe this is remedy for weak memory and a “nerve tonic” [3]. It is important to highlight that memory loss is viewed as a weakness that needs something “strong” to bring strength to the body. In this context, the bitter beverage made from *Ampelozizyphus amazonicus* is indicated, in a treatment that seeks the recovery of the body as a whole.

Concerning a possible anabolic profile, it is said that a person who takes “saracura-mirá” a lot becomes “macetona”, “parrudona” or “porruda” and “can produce a lot of children” because they will be very strong. It becomes quite evident in the reports that the person will become stout from the use of the plant, this indication being directly related to the gain in muscle mass, since the term “macetona” probably derives from the word “macete” in Portuguese, which means a mallet or hammer, capable of striking with force and impact.

The overlap between some adaptogen properties stands out. In the case of some infectious diseases, this becomes very evident, especially when the etiological agent often depends on the body’s weakness for its manifestation. An example of this occurs in the case of treatment and prevention of pneumonia and tuberculosis, since in the *quilombola* communities of Oriximiná, these diseases are associated with “weakening” [28]. In this case, it is interesting to use plants that simultaneously have an immunostimulating and toning profile, if possible, without the need to associate several plants to obtain this effect, as noted by Storey and Salem [52] and Milliken [26]. Curiously, it is noteworthy that the name “saracura” has been increasingly related to the plant’s power to “sarar” (heal) and “curar” (cure), which clearly expresses the need to prevent infectious and parasitic diseases and the need to tone the body, in a context that strengthens the whole. Finally, it is considered that rheumatism transits between these two categories (immunostimulating and toning) in an inseparable way, since it is a chronic inflammatory disease, often related to aging. The indication use for anemia can be simultaneously related to anabolic and tonic effects.

Other miscellaneous uses of *A. amazonicus* related in the literature comprise the use of the barks against snake bites [55], in the treatment of gastritis, joint pain, “women’s inflammation” and diabetes and as a revitalizer and depurative [41].

## 4. Pre-Clinical Pharmacological Studies with *A. amazonicus*

### 4.1. Pharmacological Evidence for A. amazonicus as an Adaptogen

The initial hypothesis that supports the proposal of *A. amazonicus* as an adpatogen was based on the fact that, despite the plant being used throughout the Amazon in the treatment and prevention of malaria, previous investigations of the antimalarial properties of this plant have shown that it does not have a direct action on *Plasmodium* blood stage forms, either in vivo or in red blood cell cultures [56,57]. In this way, it might be possible to suggest that the control of the infection induced by this plant could be obtained by an overall augmentation of the immunological response [56].

The immunomodulatory properties of *A. amazonicus* were demonstrated in different animal models [56,58], where the oral dose was calculated according to its traditional use [56]. The drink prepared with one tablespoon of ground bark and 200 mL of cold water affords a total solid yield of 0.21% (*w/v*) which corresponds to a 9 mg/Kg dose for an adult weighing 70 kg and taking 300 mL/day of extract. Therefore, the authors standardized the oral dose to 10 mg/kg in all biological assays [56,58].

Since several ethnobotanical studies have shown that *A. amazonicus* is useful in the treatment and prevention of malaria, the immune response of an aqueous extract from the plant was initially investigated by measuring immunoglobulin production induced by immunization with antigens in *Plasmodium*-infected mice. The plant produced a B cell response during malarial infection which led to an increase in total serum IgM and IgG and a decrease in the percentage of splenic plasma cells (CD138+ cells) in experiments run with *Plasmodium chabaudi*-infected mice pre-treated by oral administration with the plant extract for ten days [56].

The effect of *A. amazonicus* on the in vivo immune response was also investigated in an immunization model with the T-independent type 2 antigen TNP-Ficoll, which induces antigen-specific immunoglobulin production. This class of antigen stimulates B cells in the absence of T cell help and induces the production of both IgM and IgG. Oral treatment with the extract induced an increase in both IgM and IgG anti-TNP-Ficoll antibody titers. These findings suggest that components of the plant extract might have immunopotentiating effects, which were attributed in part to the saponins, which are the major components of the extract and already known to promote adjuvant properties, including the ability to enhance immunoglobulin production and also to stimulate the release of immune mediators and the proliferation of immune cells in vitro [58,59].

The potential role of *A. amazonicus* as a general immunomodulatory agent was also evaluated in another study which investigated the effects of its long-term oral treatment (10 mg/kg) on antibody production in immunized and unimmunized mice [58]. The aqueous extract from the stem bark increased the basal levels of antiovalbumin, anti-LPS and antidextran IgM antibodies, and the antidextran IgG antibodies in the unimmunized mice. No increase in antibody titers was observed after treatment in immunized mice [58]. The LPS-specific antibody levels in unimmunized SARF-treated mice were rather high when compared to the response from immunized animals. Normal individuals have basal circulating antibodies (natural antibodies, derived from early life exposure to microorganisms) even in the absence of immunization [60], which play an important role in immune regulation, homeostasis and defense against infectious diseases. Despite the finding of the ineffective role of the plant in increasing the antibody response in immunized animals, this natural product showed a remarkable effect on increasing the basal levels of the antibodies, which suggests that it is able to boost natural antibody levels and, therefore, improve host resistance [58].

Together, the above findings are in accordance with the description of plant adaptogens as compounds that increase non-specific resistance against stressors, improving one’s ability to adapt to stress.

### 4.2. Other Miscellaneous Pharmacological and Biological Activities

The pharmacological and biological effects of *A. amazonicus* have been evaluated in different models exploring the use indications of the plant. Most of them are related to the alleged antimalarial activity, for which the plant is mostly used in all Amazon regions, by different groups of traditional communities [14]. Extracts and fractions from this plant were shown to be totally inactive against blood forms of *P. falciparum* in vitro as well as in mice with experimental malaria [14,29,39]. However, the prophylactic activity of SAR has already been demonstrated, in vivo and in vitro, against the sporozoite form of contagion of malaria [54], proving this popular indication of the Amazon region [14]. On the other hand, organic extracts against *Plasmodium berghei* (schizontocidal activity, liver stage) and *Plasmodium falciparum* (3D7 and Dd2 strains, erythrocyte stage) were assessed in vitro [61]. Of the four extracts assayed against *P. berghei*, the chloroform extract showed the greatest activity (IC_50_ 30.1 μg/mL), followed by the aqueous extract (IC_50_ 39.9 μg/mL). The chloroform extract exhibited the highest antiplasmodial activity in the erythrocyte stage of *P. falciparum*, with an IC_50_ value lower than 15 μg/mL [61].

The anti-inflammatory effects of the aqueous extract from the barks from *A. amazonicus* were also evaluated to investigate whether it could alleviate the inflammatory disorders caused by malaria. Two models were tested, the formalin test and the carrageenan-induced inflammation in the subcutaneous air pouch (SAP) [56]. In the formalin model, oral administration (1, 3 and 10 mg/kg) of *A. amazonicus* stem bark aqueous extract did not show any action in the first phase (neurogenic pain response) nor in the second one (inflammatory pain response). In the SAP model, synovial inflammation is involved, caused by carrageenan injection into the air pouch that forms in the back of mice, which induces the proliferation of cells that stratify on the surface. In this model, the extract (1, 3 and 10 mg/kg) significantly reduced leukocyte migration into the SAP, in all tested doses. The protein concentration resulting from extravasation into the peritoneum was also significantly reduced, revealing the plant’s anti-inflammatory properties, which are partly due to a reduction in cell migration and are most likely due to an inhibition of the production of inflammatory mediators. These results are an indication that the plant may be effective in treating acute inflammatory disorders [56].

Given that the plant is reported as a depurative, the effects of the plant on diuresis in rats was investigated [62]. An ethanol crude extract of roots of *Ampelozizyphus amazonicus* (100–400 mg/kg) and a crude saponin mixture (50–200 mg/kg) isolated from this extract have shown antagonistic effects on diuresis in normal rats as compared to furosemide (13 mg/kg), used as positive control [62]. While the crude ethanol extract stimulates diuresis, the saponin-containing fraction induces antidiuresis. Although the tested extracts and fractions were not fully chemically characterized and do not correspond to the traditional ways of preparing the plant beverage, this effect could, at least in part, explain its popular usage as a depurative.

In a second work from the same group [63], they investigated whether atrial natriuretic peptides (ANPs) and renal ATPases play a role in the antidiuresis in rats induced by a saponin-containing fraction from the roots of this plant. As the inhibition of renal Na^+^ transport may account for the diuretic effect of different agents, they investigated the renal cortical Na^+^ pumps and renal natriuretic peptides in saponin-containing fraction-induced antidiuresis. As a result, it was shown that the saponin fraction from the roots of *A. amazonicus*, in vivo, stimulates renal ATPases and reduces the level of urine ANP. These effects are undoubtedly characteristic of an antidiuretic agent. Moreover, the saponin fraction abolished the diuretic effect of furosemide, a classical diuretic that affects renal Na^+^-ATPases [63].

### 4.3. Safety and Toxicity Issues

Literature data recording safety and toxicity issues about *A. amazonicus* are scarce. One study [56] investigated the acute toxicity of a single dose oral administration (10 mg/kg) of an aqueous extract from the bark. Behavior parameters included convulsion, hyperactivity, sedation, grooming, loss of righting reflex, increased or decreased respiration and food and water intake over a period of 15 days. No behavioral alterations, lesions or gastric bleeding was observed. Additionally, no signs of intoxication, including convulsion, death or gastric ulcer, were observed. It is noteworthy that the oral administration of the plant extract did not show any gastric disorders because saponin-containing plant extracts can cause irritation of the gastric mucosa as an important side effect [56].

In another study designed to evaluate the immunomodulatory actions of an aqueous extract from bark, the effects of its long-term oral treatment (10 mg/kg) in mice were observed [58]. Since the plant has a high content of saponin, the authors tested whether 12 days of oral treatment would alter circulating red blood cell levels in mice, and no alteration was observed.

Despite the limited number of papers covering toxicology studies for this plant, the long tradition of its use, documented for over 30 years, has been accepted in Brazil for the registration of herbal medicines based on traditionality, within well-established criteria, as established in the Brazilian resolution RDC 26/2014 [64]. In this sense, it is possible to find records of the use of “saracura-mirá” as a medicinal plant in at least three books from the 1970s and 1980s [31,34,35].

## 5. Present Status of the Market, Supply Chains, Intellectual Property and Biotechnological Studies

**Figure 3:**
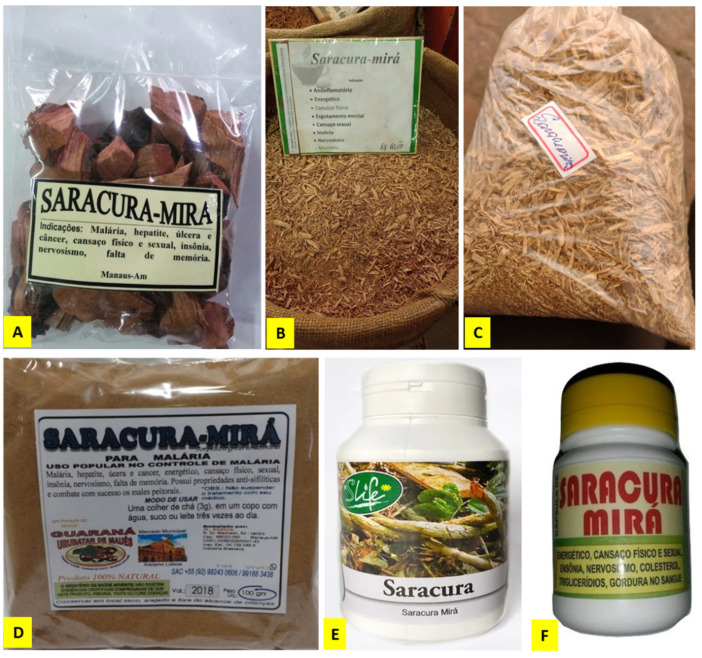
Commercial samples of “saracura-mirá” sold in open markets in northern cities of Brazil and on some internet websites. (**A**) Stem bark sold in pieces; (**B**,**C**) stem bark comminuted in small fragments with part of the wood sold at the Ver-O-Peso Market, Belém, Pará State, Brazil; (**D**) powdered bark; (**E**,**F**) irregular products (unregistered at the Brazilian National Health Surveillance Agency, ANVISA) sold as capsules (source: https://produto.mercadolivre.com.br/MLB-1909499730-saracura-kit-12-frascos-100-natural-60-capsulas-de-500mg-, accessed 31 October 2021).

It is possible to find the plant in traditional/popular markets in the Brazilian Amazon regions, but, as far as we know, there is no established supply chain. Currently, the plant is available as a raw material in medicinal plant open markets in northern cities in Brazil, sold in natura, in the form of chips or small pieces, usually dry, as well as in the form of irregular products such as capsules, etc. (Figure 3). In smaller towns and communities, it is usually collected straight from the forest, and prepared directly before use, as shown in Figure 2. Although the market is apparently very restricted and local, two patents were found in relation to this plant species. One, Brazilian, granted in 2020 (BR 102014020896-8 A2), is related to a reconstitutable powder composition comprising the spray dried powder extract of *A. amazonicus*, microencapsulated with or without maltodextrin, mandarin pulp, citric acid and sugar. The patent is also for the use of said composition to manufacture an adaptogenic drink, which may be produced by reconstitution of the composition in water. The other is a Japanese patent of the plant for a skin care preparation issued in 2002 (JP2002308750 23/10/2002), whose claims are: “medication to quickly increase the skin’s protection against UV rays, preventing inflammation, hyperemia or darkening of the skin after sunburn”. The agent that promotes this protection is taken from a series of plants including *Ampelozizyphus amazonicus* [65].

Although there is no product registered in the Brazilian (or international) pharmaceutical market based on *A. amazonicus* extracts, there is a study on the biotechnological properties of a stem bark dry extract obtained by spray drying. The powder properties, such as the particle morphology, size distribution, solubility and wettability, were analyzed [58], showing that the extract could be efficiently dried without drying adjuvants. This can be considered an advantage since the addition of such additives in large amounts increases the production cost and may also alter the original flavor of the final product, thus risking consumer disapproval [58]. The particles tended to have a spherical shape and a unimodal size distribution, and they also had good rehydration characteristics and high saponin content (33%).

Due to the growing interest in dietary supplements with adaptogen properties, these data show that this plant represents a good possibility of technological development for pharmaceutical products or food supplements/nutraceuticals.

## 6. Chemistry of *Ampelozizyphus*

**Figure 4:**
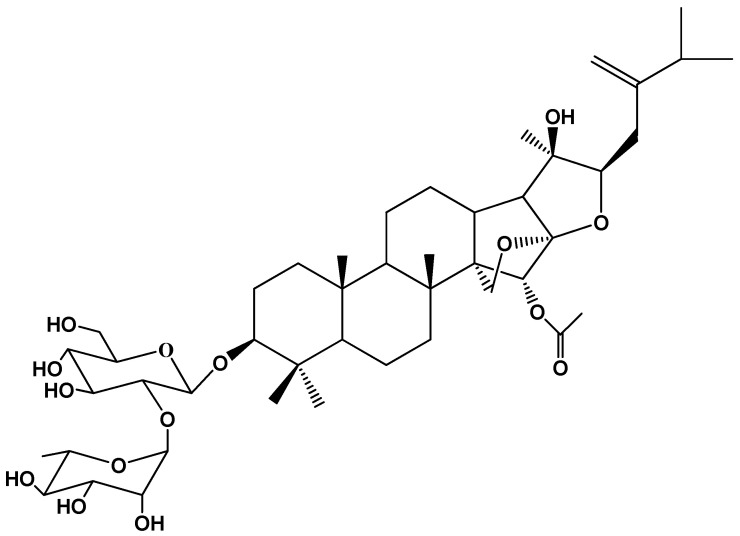
Chemical structure of ampelozizyphoside A.

The chemistry of *A. amazonicus* follows the trend of other Rhamnaceae representatives, such as the *Ziziphus* genus (*Z. joazeiro*, *Z. jujuba*, *Z. spina-christi*, etc.), which is the production triterpenes and saponins, especially those of the dammarane skeleton [66,67,68]. The first works on the chemistry of this plant are from Brandão [55,69] who described in the 1990s the isolation and identification of three saponins with a dammarane-type aglycone from the roots of the plant. In 1993 [69], the group described the new saponin ampelozizyphoside A, with an undescribed dammarane aglycone skeleton bearing 31 carbon atoms, named ampelozigenin [69] (Figure 4). Then, approximately 20 years passed with a gap in the literature on the chemistry of this plant, with sparse works reporting the content of saponins, without further details about their structures. Additionally, the first studies on the chemistry of this plant were conducted on the roots. However, the stem bark of the plant, besides being the most used, is richer in saponin content as revealed by Amaral and coworkers [20]. The reported content of saponins for the root barks is 48% [70].

The high saponin content in the root and stem bark of the plant explains most of its medicinal properties, but free triterpenes are also described: melaleucic acid, 3*β*,27*α*-dihydroxylup-20(29)-en-28*β*-oic acid, betulinic acid, betulin, lupeol and phytosteroids [55,69,71].

**Figure 5:**
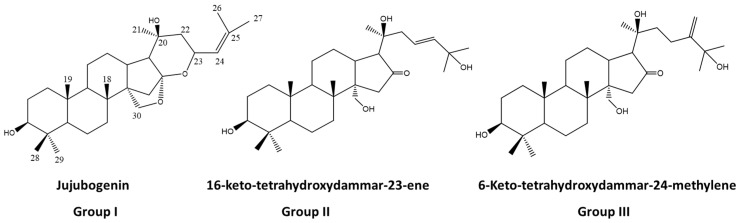
Main aglycones of saponins from *Ampelozizyphus amazonicus*.

More recently, some works have unveiled the complex saponin profile of aqueous extracts from the bark and wood of this plant. Preliminary chemical analysis by high-performance liquid chromatography coupled to ESI-MS/MS experiments with the aqueous extract from the stem bark of the plant showed a complex saponin profile with deprotonated molecular [M-H]^−^ ions in the range of m/z 800–1000 Da, where the presence of free triterpenes was also evidenced [56]. This is an important finding, since these terpenoids are normally not water soluble, but due to the high content of saponins in the plant, they are delivered to the water in the extraction process. The saponins of an aqueous extract from the stem bark of the plant, prepared by a company specialized in the preparation of botanical extracts, were also characterized by ultra-high-performance liquid chromatography/high-resolution accurate mass spectrometry (HPLC-HRMS^n^). It revealed at least three main groups of dammarane saponin aglycones represented by the structures in Figure 5—group I, having the jujubogenin aglycone skeleton, and groups II and III having two newly proposed 16-keto-dammarane skeleton types, the third one being a C-31 saponin aglycone, with a 24-methylene group [58]. The proposal of the C-31 aglycone was possible based on the work of Brandão and coworkers, that published in 1993 the isolation of ampelozizyphoside A, with a dammarane aglycone skeleton type with 31 carbon atoms, named ampelozigenin [69].

Later, Figueiredo and coworkers [72] revealed the occurrence of triterpene saponins bearing a pentacyclic triterpene aglycone. Due to the complex nature of the aqueous extracts of this plant, countercurrent chromatography studies were undertaken, leading to the separation of some of the saponins by skeleton type, separating the oleanane from the dammarane types, affording simpler fractions that were then analyzed by LC coupled to high-resolution mass spectrometry [72]. This study expanded the number and type of structures identified in the plant to twenty-eight different ones.

**Figure 6:**
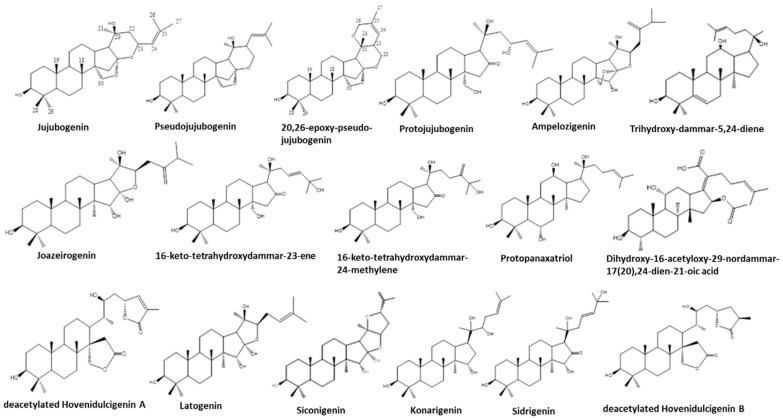
Newly found [66] aglycones of saponins from *Ampelozizyphus amazonicus*.

More recently, the chemical profiling and tentative structural identification of the major saponins present in aqueous and ethanol extracts of bark and wood of the plant were accomplished by mass spectrometry [66]. In the traditional process of beverage preparation, the bark is removed, and the wood discarded. However, considering that the plant is apparently not cultivated, and the possibility of overharvesting and overexploitation that could lead to serious decline of its natural populations, studies were undertaken to investigate the saponin profile of the wood extract to provide a sustainable and less wasteful use of the plant material. The study revealed a high chemical complexity for the wood extract that is comparable to that of the bark. In total, 95 saponins were tentatively identified in *A. amazonicus* wood and bark, including 73 which were described for the first time as tentative structures for the plant species [66]. At least 17 different sapogenin skeletons, bearing three and four sugar residues, as well as ester residues, were proposed (Figure 6).

## 7. Perspectives and Conclusions

In this review, the ethnopharmacological and/or ethnobotanical literature data on *Ampelozizyphus amazonicus* were gathered, with a special focus on this plant’s use that can be connected to the concept of adaptogens, which were interpreted in the light of the adaptogen concept. The compilation of the literature is important to understand the state of the art, and to demonstrate the traditional uses of the plant, related to the effectiveness of the plant demonstrated by its continuous use, which is the basis to support the further development of traditional herbal products. In this sense, this review shows unambiguously that *Ampelozizyphus amazonicus*, known as “saracura-mirá”, could act as an adaptogen plant. The ethnobotanical literature data survey shows a broad adaptogen profile of *A. amazonicus*, so as to propose it as an “Amazonian ginseng”, since the plant could be categorized in all adaptogen profiles—immunostimulant, nootropic, anabolic, tonic and geriatric agents. Pharmacological assays designed to evaluate the immunomodulatory properties of the plant demonstrated that an extract prepared according to the traditional method produces a remarkable effect on increasing the basal levels of antibodies, which suggests that the plant may be able to boost natural antibody levels and, therefore, improve host resistance. These findings are in accordance with the description of plant adaptogens that are compounds that increase non-specific resistance against stressors, improving one’s ability to adapt to stress. These observations are also consistent with the popular use of the extract as a stimulant, energetic and fortifying tonic.

In view of a current market trend of looking for alternative sources for functional foods, the adaptogen effect of *A. amazonicus* could be an interesting attribute to consider for the use of this plant in the formulation of foods or pharmaceuticals.

In addition, this review brings current agronomic, chemical, pharmacological, toxicological and marketing data. The literature review showed that the plant is not being cultivated, and a detailed survey on the natural populations of this plant is needed, as well as agronomical studies, should the plant be exploited more deeply to furnish the market with the bark as a raw material for pharmaceutical or nutraceutical purposes. Another important gap is the lack of well-developed quality control methods to ensure the raw material’s quality, for instance, to avoid the risk of its replacement by other woody vines, with reddish bark and white wood. Simple and fast quality control methods for evaluating the quality of extracts so as to generate fingerprints are also needed, in order to build a complete pharmacopeic monography.

## Figures and Tables

**Table 1:** Use indications of *Ampelozizyphus amazonicus* correlated with adaptogen properties in the literature.

Adaptogen Profile **	Use Indications (Number of References that Cite Each Use Indication)	References
Immunostimulants (49)	treat malaria (16) *; prevent malaria (5) *; fever/febrile fever (5); chill (2); flu/cold (2); prevent disease (1); against infectious and parasitic diseases (1); “angry wound”/leishmaniasis (1); diarrhea (2); intestinal infection (1); sexually transmitted diseases (STDs) (1); verminosis (1); COVID-19 (1); inflammation in general (3); general pain/body pain (5); women’s inflammation (1); hemorrhoid (1)	[11,14,25,26,27,28,29,30,31,32,33,34,35,36,37,38,39,40,41,42,43,44,45,46,47,48,49]
Tonic and Geriatric Agents (32)	aphrodisiac (3); energetic/giving energy (2); tonic in general (1); antifatigue (1); stimulant (1); rejuvenating (1); revitalizing (2); indisposition (1); before working (1); appetizing (lack of appetite) (1); liver (or liver) disorders (5); gastrointestinal disorders (3); cholesterol (1); diabetes (2); kidney diseases (1); diuretic (1); purgative (1); well-being (1); insomnia/sleep disorders (2)	[14,24,27,40,41,42,43,50]
Nootropics (2)	nerve tonic (1); poor memory (1)	[3,28]
Anabolic Agents (3)	to become strong (2); fortifier (1)	[27,29,51]
Immunostimulant/Tonic and Geriatric Agents (18)	depurative (6); blood purifying (1); tuberculosis/severe cough/severe cough with blood (2); pneumonia (1); restoration of health (1); rheumatism (1); prostate inflammation (1); gastritis/stomach pain (2); intoxication (2); malaise due to alcohol abuse or fatty foods (1)	[26,27,29,35,41,44,52]
Anabolic/Tonic and Geriatric Agents (2)	anemia (2)	[3,41]

* Considered as malaria citations of tertian fever, malaria fever and impaludism. ** Total number of use indications by citations for each adaptogenic category, considering the literature surveyed.

## Data Availability

Not applicable.
